# Neurofibromatosis type 1 associated with papillary thyroid carcinoma incidentally detected by thyroid ultrasonography: a case report

**DOI:** 10.1186/1752-1947-6-179

**Published:** 2012-07-02

**Authors:** Bu Kyung Kim, Young Sik Choi, Sangeon Gwoo, Yo Han Park, Song I Yang, Jeong Hoon Kim

**Affiliations:** 1 Department of Internal Medicine, Kosin University College of Medicine, 262 Gamcheon Street SeoGu, Busan, 602-703, South Korea; 2 Department of General Surgery, Kosin University College of Medicine, 262 Gamcheon Street SeoGu, Busan, 602-703, South Korea

## Abstract

**Introduction:**

Neurofibromatosis type 1 is a common heritable neurocutaneous disorder. Neurofibromatosis type 1 may be associated with tumors of the central nervous system and pheochromocytoma. However, papillary thyroid carcinoma associated with neurofibromatosis type 1 is very rare. We present what is, to the best of our knowledge, the first case of papillary thyroid carcinoma to be detected incidentally by ultrasonography in a patient with neurofibromatosis type 1.

**Case presentation:**

A 63-year-old South Korean man with neurofibromatosis type 1 presented to our study hospital because of thyroid nodules detected incidentally by ultrasonography. Papillary thyroid carcinoma was diagnosed by ultrasonography-guided fine-needle aspiration, and then a total thyroidectomy with central compartment neck dissection was performed. The B isoform of the Raf^V600E^ mutation was identified by multiplex real-time polymerase chain reaction assay.

**Conclusions:**

Papillary thyroid carcinoma associated with neurofibromatosis type 1 is very rare. However, it is speculated that papillary thyroid carcinoma is more likely to be detected in patients with neurofibromatosis type 1 if screening by ultrasonography is performed for them.

## Introduction

Neurofibromatosis type 1 (NF1) is an autosomal dominant neurocutaneous disorder in which tumors involving the sheaths or peripheral nerves are associated with café-au-lait spots. NF1 is caused by mutation of the NF1 gene on chromosome 17q11.2 [1]. The NF1 gene encodes for neurofibromin, which acts as a tumor-suppressor protein. NF1 may be associated with other tumors of the central nervous system, including optic glioma, glioblastoma, and meningioma, and rarely with pheochromocytoma. However, papillary thyroid carcinoma (PTC) associated with NF1 is very rare, and only three cases – all of which involved large tumors – have been reported in the literature [2-4]. Recently, the PTC of a patient with NF1 was diagnosed incidentally by thyroid ultrasonography (US). To the best of our knowledge, it is the first case of PTC to be detected incidentally by US in a patient with NF1.

## Case presentation

**Figure 1:**
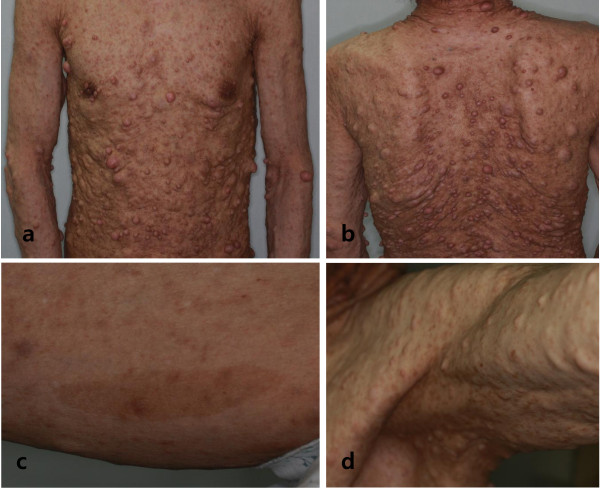
**(a,b) Neurofibromas covering the entire surface of the patient’s body.** (**c**) Café-au-lait macules on the calf. (**d**) Skin fold freckling.

A 63-year-old South Korean man with NF1 presented at our endocrine center because of thyroid nodules that were found incidentally by US at a general health checkup. His family history was unremarkable. A physical examination revealed neurofibromas covering the entire surface of his body, café-au-lait macules on his calf, and skin fold freckling (Figure 1); there were no Lisch nodules on his iris, and his thyroid was unremarkable. The results of a complete blood count, serum biochemistry, and urine analysis were normal. Our patient was euthyroid and had serum levels of free Thyroxine (T4) at 0.83ng/dL (normal range was 0.78 to 1.54ng/dL), thyroid-stimulating hormone levels of 3.495μIU/mL (normal range was 0.55 to 4.78μIU/mL), and thyroglobulin of 43.05ng/mL (normal range was 1.4 to 78.0ng/mL). Autoimmune antibodies such as anti-thyroglobulin and anti-microsomal antibodies were within the normal limits.

**Figure 2:**
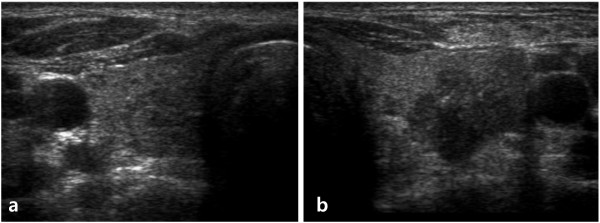
**Transverse ultrasonography images of thyroid nodules.** (**a**) In the right lobe, a 0.79 × 0.75cm hypoechoic and homogeneous nodule with a halo was observed. (**b**) In the left lobe, a markedly hypoechoic 1.25 × 1.38cm nodule with irregular margins, microcalcification, and extracapsullar extension was observed in the middle portion.

Thyroid US showed a 0.79 × 0.75cm hypoechoic, homogeneous nodule with clear margins in the right lobe (Figure 2a) and a 1.25 × 1.38cm markedly hypoechoic, microcalcified, extracapsular extension nodule with irregular margins in the mid-portion of the left lobe (Figure 2b). A US-guided fine-needle aspiration biopsy (FNAB) was performed on the thyroid nodule. FNA cytology diagnosed suspicious PTC. Magnetic resonance imaging of the brain did not indicate hydrocephalus. Our patient underwent a total thyroidectomy with a central compartment neck dissection for the thyroid tumor. Metastasis of seven central lymph nodes at left level VI was confirmed. The tumor in the left thyroid gland was gray-white and abutted the thyroid capsule. Microscopically, the left thyroid tumor mass was dense and contained pink-staining colloid, clear overlapping nuclei, and nuclear grooves. The left thyroid tumor was diagnosed as PTC. The nodule in the right thyroid gland was microscopically diagnosed as nodular hyperplasia.

A solid skin tumor was visible as a gray-white round mass. The outer surface was surrounded by a thin capsule, and the cut surface was homogenously gray-white. A microscopic examination showed that this tumor consisted of spindle-shaped cells with wavy and tapered nuclei. The cells showed positive staining for S-100, and a neurofibroma was diagnosed.

**Figure 3:**
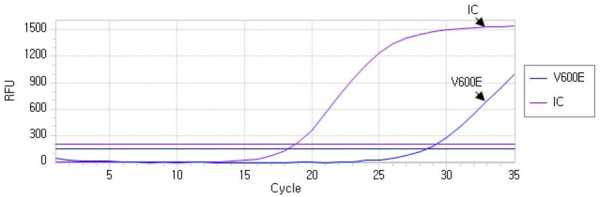
**Detection of V600E mutation by using Anyplex*™ BRAFV600E*Real-time Detection (Seegene).** This product allows simultaneous amplification of total nucleic acid of V600E and internal control (human beta globin gene).

B isoform of the Raf (BRAF)^V600E^ mutation was identified by multiplex real-time polymerase chain reaction (PCR) assay by using Anyplex™ *BRAF V600E* Real-time Detection (version 2.0; Seegene, Seoul, South Korea), which allows simultaneous amplification of total nucleic acid of V600E mutation of BRAF and internal control (human beta globin gene) (Figure 3). Genomic deoxyribonucleic acid (DNA) was isolated from the formalin-fixed paraffin-embedded (FFPE) surgical tissue resections by using a QIAamp® DNA FFPE tissue kit (Qiagen, Hilden, Germany), and real-time PCR was performed with the CFX96™ Real-Time PCR system (Bio-Rad Laboratories, Inc., Hercules, CA, USA).

At present, our patient is following up as an out-patient. Radioactive iodine treatment is planned for adjuvant therapy of the papillary carcinoma.

## Discussion

NF1, also known as von Recklinghausen disease, is an autosomal dominant multisystem disorder that affects approximately 1 in 3500 people [5]. In 1987, seven cardinal diagnostic criteria for NF1 were established [6]. If any two of the following seven criteria are met, a diagnosis of NF1 is made: (a) two or more neurofibromas on or under the skin or one plexiform neurofibroma, (b) freckling of the groin or the axilla (arm pit), (c) six or more café-au-lait spots measuring 5mm in the greatest diameter in prepubescent individuals and over 15mm in the greatest diameter in post-pubescent individuals, (d) skeletal abnormalities such as sphenoid dysplasia or thinning of the cortex of the long bones of the body, (e) two or more Lisch nodules (hamartomas of the iris), (f) optic glioma, or (g) a first-degree relative with NF1. These diagnostic criteria are highly specific to adults with NF1. The patient in this study presented with neurofibromas all over the surface of his body, multiple café-au-lait spots, and axillary freckling. The NF1 gene was discovered in 1990. Mutational analysis is now available in a clinical setting and is useful for diagnostic confirmation of individuals who do not fulfill diagnostic criteria or when a prenatal diagnosis is desired [7].

Neurofibromatosis is an autosomal dominant disorder, meaning that only one copy of the affected gene is needed for the disorder to develop. Therefore, if only one parent has neurofibromatosis, his or her children have a 50% chance of developing the condition as well. The severity in affected individuals can vary and this variation may be due to variable expressivity. An individual with mild clinical symptoms can have a more severe phenotype and so genetic counseling is important [7]. Approximately half of the known cases are due to *de novo* mutations and no other affected family members are seen. The patient in this case had no family history of NF1, suggesting that he has a *de novo* mutation.

PTC is one of the most common types of endocrine cancer. The prevalence of thyroid cancer is rapidly increasing in South Korea, and most cases are PTC. The high rates of incidence of RET/PTC rearrangements or point mutations in RAS and c-MET oncogenes are genetic hallmarks of PTC [3]. The BRAF kinase is a serine-threonine kinase that mediates signal transduction through the MEK-ERK pathway. An activating mutation of the BRAF kinase gene, located on exon 15, was recently found to result in a valine-to-glutamic acid substitution at amino acid 600 (*BRAF*^*V600E*^ mutation) that is an oncogene in human cancer and the most common mutation in PTC [8]. In recent years, the *BRAF*^*V600E*^ mutation has shown a high specificity for PTC, and its prevalence is highly variable, ranging from 30% to more than 80%, depending on the study. In the present study, the *BRAF*^*V600E*^ mutation was identified by multiplex real-time PCR. This method is as sensitive as dual-priming oligonucleotide-based multiplex PCR (Seegene) for detecting BRAF^V600E^ mutations.

The NF1 gene located on chromosome 17q11.2 encodes neurofibromin. The Ras-GAP is a potentially functional domain of neurofibromin [9]. The Ras-GAP-related domain (Ras-GRD) accelerates the conversion of active Ras-GTP to inactive Ras-GDP in various cell types and acts as a negative regulator of the p21ras signaling pathway [10]. Ras GTPases interact with multiple pathways, including the RAF-MEK-ERK mitogen-activated protein kinase pathway. Mutations in the NF1 gene result in abnormal cell growth and in the formation of benign and malignant tumors [3]. Because *BRAF*^*V600E*^ mutation and NF1 gene mutation are both involved in the MEK-ERK pathway, Koksal *et al.*[3] suggested that the development of PTC in patients with NF1 may be associated with the ras gene but that further evidence is necessary to confirm this association.

PTCs are very rare in NF1. Only three cases have been reported. Nakamura *et al.*[4] reported a case of NF1 associated with a pheochromocytoma and PTC in a 58-year-old woman. Hashiba *et al.*[2] reported a case of skull metastasis from PTC in a 74-year-old woman with NF1. Koksal *et al.*[3] reported neurofibroma adjacent to the thyroid gland and PTC in a patient with NF1. These cases all involved large tumors. The nodule in this study was small and was incidentally diagnosed by thyroid US. US features of malignant nodules include the presence of microcalcifications, hypoechogenicity, and irregular margins and the absence of a halo, predominantly solid composition, and intra-nodular vascularity [11]. The nodule in this case had irregular margins, hypoechogenicity, microcalcifications, and extracapsular extension on US. This nodule was suspected to be associated with PTC, and a US-guided FNAB was performed at the thyroid nodule. The diagnosis based on FNA cytology indicated suspicious PTC. A total thyroidectomy with central compartment neck dissection of the thyroid tumor was performed. The tumor in the left thyroid lobe was diagnosed as PTC, and metastasis of seven central lymph nodes at left level VI was confirmed.

## Conclusions

NF1 is a common heritable neurocutaneous disorder. However, PTC associated with NF1 is very rare and only three cases have been reported. To the best of our knowledge, this is the first case of PTC to be detected incidentally by US in a patient with NF1. It is speculated that PTC is more likely to be detected in patients with NF1 if screening by ultrasound is performed.

## Consent

Written informed consent was obtained from the patient for publication of this case report and any accompanying images. A copy of the written consent is available for review by the Editor-in-Chief of this journal.

## Abbreviations

BRAF, B isoform of the Raf; DNA, deoxyribonucleic acid; FFPE, formalin-fixed paraffin-embedded; FNA, fine-needle aspiration; FNAB, fine-needle aspiration biopsy; NFI, neurofibromatosis type 1; PCR, polymerase chain reaction; PTC, papillary thyroid carcinoma; US, ultrasonography.

## Competing interests

The authors declare that they have no competing interests.

## Authors’ contributions

BK, YC, SG, and YP interpreted patient data, collected previous published literature on the subject, and helped to write the manuscript. SY and JK performed the surgical procedure and helped to write the manuscript. All authors read and approved the final manuscript.
